# Combination of CD34-positive cell subsets with infarcted myocardium-like matrix stiffness: a potential solution to cell-based cardiac repair

**DOI:** 10.1111/jcmm.12301

**Published:** 2014-06-19

**Authors:** Shuning Zhang, Xin Ma, Kang Yao, Hong Zhu, Zheyong Huang, Li Shen, Juying Qian, Yunzeng Zou, Aijun Sun, Junbo Ge

**Affiliations:** a Shanghai Institute of Cardiovascular Diseases, Zhongshan Hospital, Fudan UniversityShanghai, China; b Institutes of Biomedical Sciences, Fudan UniversityShanghai, China

**Keywords:** matrix stiffness, myocardial infarction, CD34-positive cells, endothelial cell lineage

## Abstract

Detection of the optimal cell transplantation strategy for myocardial infarction (MI) has attracted a great deal of attention. Commitment of engrafted cells to angiogenesis within damaged myocardium is regarded as one of the major targets in cell-based cardiac repair. Bone marrow–derived CD34-positive cells, a well-characterized population of stem cells, might represent highly functional endothelial progenitor cells and result in the formation of new blood vessels. Recently, physical microenvironment (extracellular matrix stiffness) around the engrafted cells was found to exert an essential impact on their fate. Stem cells are able to feel and respond to the tissue-like matrix stiffness to commit to a relevant lineage. Notably, the infarct area after MI experiences a time-dependent stiffness change from flexible to rigid. Our previous observations demonstrated myocardial stiffness-dependent differentiation of the unselected bone marrow–derived mononuclear cells (BMMNCs) along endothelial lineage cells. Myocardial stiffness (∽42 kPa) within the optimal time domain of cell engraftment (at week 1 to 2) after MI provided a more favourable physical microenvironment for cell specification and cell-based cardiac repair. However, the difference in tissue stiffness-dependent cell differentiation between the specific cell subsets expressing and no expressing CD34 phenotype remains uncertain. We presumed that CD34-positive cell subsets facilitated angiogenesis and subsequently resulted in cardiac repair under induction of infarcted myocardium-like matrix stiffness compared with CD34-negative cells. If the hypothesis were true, it would contribute greatly to detect the optimal cell subsets for cell therapy and to establish an optimized therapy strategy for cell-based cardiac repair.

The net loss of cardiomyocytes and the massive deposition of extracellular matrix following MI are the key factors resulting in the remodelling and in the impairment of cardiac-pump function [1]. Vascular regeneration with stem or progenitor cells is one of the promising therapeutic strategies for myocardial repair in ischaemic cardiovascular diseases [2]. Neovascularization or angiogenesis may improve cardiac function and prevent further scar tissue formation following ischaemia-induced myocardial damage. Cell therapy, as an effective treatment strategy to induce neovascularization within the infarcted myocardium and to reverse myocardial remodelling, is an attractive option for cardiac repair after MI [3,4]. Bone marrow–derived cells, particularly mononuclear cells, are known to exhibit angiogenic potential both *in vitro* and *in vivo* [2] and are now widely used in clinical studies to attenuate infarct size and improve cardiac function after MI [5]. Notably, BMMNCs represented an unselected and non-specific cell lineage. Among them, only ∽1% express a stem cell phenotype [6].

CD34 cell-surface antigen is first characterized as a protein by identifying multipotent haematopoietic progenitor cells. Moreover, a novel type of interstitial cell expressing CD34 antigen (CD34-positive) very recently discovered in various organs, defined as ‘telocytes’, has been verified to be involved in neo-angiogenesis after tissue damage [7]. Likewise, CD34-positive mononuclear cells derived from bone marrow, a well-characterized population of stem cells, might represent highly functional endothelial progenitor cells [8] and have been shown to contribute to the formation of new blood vessels in animal ischaemia model [9,10]. The belief that the CD34-positive mononuclear cells promote therapeutic angiogenesis arose primarily from the fact that both ‘endothelial progenitor cells’ and fully differentiated endothelial cells were found to express the CD34 antigen [11]. A growing body of evidence indicated that CD34-positive cells have the ability to differentiate into endothelial lineage cells and present potent angiogenic properties [8,12]. Unlike this, CD34-positive telocytes cells might participate in neo-angiogenesis through the direct (physical) contact with endothelial tubes, as well as the indirect (chemical) positive influence within the angiogenic zones [13,14]. However, the optimal factors that promote specification of CD34-positive cells remain to be elucidated. Various niche factors interact with stem cells to regulate cell fate [15,16]. Traditionally, several biochemical factors, especially VEGF, are known to be essential for the *in vitro* differentiation of purified CD34-positive cells into endothelial lineage cells [17]. This observation is consistent with *in vivo* studies demonstrating the importance of VEGF in vasculogenesis. In addition, physical characteristics (referring to matrix stiffness) of extracellular matrix, providing structural and biochemical support to the surrounding cells [18,19], have been verified to determine the fate of several stem cells [20,21]. Recently, a serial of researches begin to focus on the effect of the physical microenvironment around the engrafted cells on their specification. The stiffness of extracellular matrix corresponding to specific tissues could promote tissue-mimetic differentiation of stem cells *in vitro* [22]. The cellular phenotype and behaviour after differentiation induced by deformable matrix with varied stiffness may more closely mimic that of the cells in their normal host tissue.

Interestingly, the infarct area after MI experiences a time-dependent stiffness change from flexible to rigid [23]. It might result from eosinophil infiltration, the accumulation of fibroblasts or myofibroblast, and abundant deposits of collagen fibrils at the various stages after MI [7]. It is natural to associate optimal timing of cell transplantation in cardiac repair (1–2 weeks after MI) [24,25] with the time-dependent change in physical microenvironment following MI. Myocardial stiffness within this optimal time frame might be more suitable for the phenotypic plasticity and functional specification of the engrafted cells along some beneficial cell lineages, such as endothelial cells, than that at others time-points [26]. Indeed, in our previous experimental research, we verified our presumption by conducting BMMNCs culture [27]. The results demonstrated that the optimal efficacy of cell therapy at 1–2 weeks after MI seemed likely to result from non-VEGF dependent angiogenesis, and myocardial stiffness at this time domains was more suitable for the specification of implanted cells along endothelial lineage cells [27]. The role of the specific cells population expressing CD34 surface phenotype with stem cell characteristics among BMMNCs in tissue stiffness–dependent cell-based cardiac repair remains uncertain.

On the basis of the above scientific findings, it has been suggested that among BMMNCs, CD34-positive mononuclear cell population might be easily induced to differentiate into endothelial cell lineage, which facilitated angiogenesis as well as resulted in cardiac repair and amelioration of cardiac functions, by flexible matrix with infarcted myocardium-like stiffness in comparison with CD34-negative subsets. Moreover, CD34-positive cells under induction of matrix stiffness might present a similar stiffness-dependent differentiation principle as BMMNCs and exert an essential impact on the efficacy of cell implantation for damaged myocardium.

We have performed a preliminary *in vitro* experiment to test the scientific hypothesis. Polyacrylamide gel substrates with varied stiffness were used to mechanically mimic the infarcted myocardium as described in our previous research [27]. Bone marrow–derived CD34-positive and -negative mononuclear cells, which were separated from murine BMMNCs by magnetic-activated cell sorting technique, respectively, were cultured with the same cell number of 5 × 10^5^ per well in the flexible culture substrates under the lower concentrations of VEGF of 2.5 ng/ml. Endothelial lineage commitment of CD34-positive cells and CD34-negative cells was identified by immunofluorescent technique with laser scanning confocal microscope respectively. The pilot findings demonstrated that, regardless of culture substrate stiffness, CD34-positive cell culture system consistently presented the higher percentage and the much more number of double-positive cells for DiI-labelled acetylated low-density lipoprotein uptake and FITC-labelled ulex europaeus agglutinin I lectin (FITC-UEA-1) binding than CD 34-negative system. Meanwhile, a more double-positive cell number was shown in the stiffness of 42 kPa, which mechanically mimics stiffness of infarcted myocardium between 1 and 2 weeks following MI, compared with other flexibility. Overall, combination of CD34-positive cell subsets with tissue-like matrix stiffness might be a potential solution to cell-based cardiac repair following MI.

If the present hypothesis were true, it will contribute greatly to understand the potential optimal cell type for cell transplantation and to establish an optimized treatment strategy for cell-based cardiac repair. Moreover, the identification of the optimal cell subsets will offer considerable help to elucidate the potential mechanism of cell specification along favourable cell lineage and cell transplantation therapy through promoting angiogenesis or neovasculogenesis within damaged myocardium. In addition, both implanted cell type and mechanical microenvironments should be paid greater attention to in cell therapy and tissue engineering. The combined strategy would offer the more therapeutic potential for the preservation or recovery of myocardial tissue integrity and function after MI.
